# The impact on quality of life from informing diagnosis in patients with cancer: a systematic review and meta-analysis

**DOI:** 10.1186/s12885-020-07096-6

**Published:** 2020-07-02

**Authors:** Miao Wan, Xianggui Luo, Juan Wang, Louis. B Mvogo Ndzana, Chen Chang, Zhenfen Li, Jianglin Zhang

**Affiliations:** 1 Dermatology Department of Xiangya Hospital, Central SouthUniversity, No.87, Xiangya Road, Kaifu District, Changsha, 410000 Hunan Province China; 2 Maternity Department of Xiangya Hospital, Central South University, Lanzhou, 730000 China; 3 The Second Clinical Medical College of Lanzhou University, Lanzhou, 730000 China

**Keywords:** Diagnosis awareness, Cancer, Diagnosis disclosure, Meta-analysis, Quality of life, Systematic review

## Abstract

**Background:**

The aim of this study was to assess the impact on quality of life from informing patients with cancer of their diagnosis and disease status.

**Method:**

We searched the follow databases, PubMed, CENTRAL (Cochrane Central Register of Controlled Trials), PsycINFO, WEB OF SCIENCE, Embase, CBM (Chinese Biomedical Literature database), WANFANG database (Chinese Medicine Premier), and CNKI (China National Knowledge Infrastructure), using the following terms: *neoplasm, cancer, tumor, tumor, carcinoma, disclosure, truth telling, breaking bad news, knowledge, knowing, awareness, quality of life, QOL.* Pairs of reviewers independently screened documents and extracted the data, and the meta-analysis was performed using Revman 5.0 software.

**Results:**

Eleven thousand seven hundred forty records retrieved from the databases and 23 studies were included in the final analysis. A meta-analysis revealed that there were no differences in either the general quality of life and symptoms of fatigue, pain, dyspnea, insomnia, appetite loss, and diarrhea, between informed and uniformed cancer patients (*P* > 0.05). There were also no differences found between the patient groups in physical function, role function, cognitive activity, and emotional function (*P* > 0.05). In terms of vitality, patients who were completely informed about their diagnosis showed higher vitality than uniformed patients. Uninformed patients seemed to have lower social function scores. Between partly informed and uninformed cancer patients, no differences were found in their general quality of life, function domains, and disease-related symptoms (*P* > 0.05).

**Conclusion:**

Informing cancer patients of their diagnosis may not have a detrimental effect on their quality of life.

**Trial registration:**

CRD42017060073.

## Background

In 2015, an estimated 17.5 million new cancer cases and 8.8 million cancer deaths occurred worldwide [1]. Health care providers are usually reluctant to inform their patients of a cancer diagnosis [2, 3] and although it is ethical to inform patients of their diagnosis and disease status, plenty of physicians and patients’ relatives still believe that concealing diagnosis and disease status was significant for a patients’ prognosis.

Many researchers are also interested in this topic and one study showed that patients’ awareness of disease status significantly increased rates of psychiatric disorders, such as depression and anxiety [4]. Conversely, another study showed that patient awareness of disease status helped to decrease the occurrence of depression and anxiety in patients with end-of-life cancer [5]. A systematic review in 2015 tried to confirm the influence of disease status awareness on the quality of life of patients with metastatic cancer, however, only mixed findings were found on the association [6]. There has been no systematic review with meta-analysis to assess the impact of awareness of diagnosis on quality of life (QoL) for patients with cancer.

In this review, we have systematically collected and reviewed studies focusing on the association between diagnosis disclosure and QoL in cancer patients, and have conducted a meta-analysis to quantitatively present this association by pooling effect estimates.

## Methods

### Inclusion and exclusion criteria

The following inclusion criteria were used to optimize selection of appropriate articles: articles needed to (1) be written in either English or Chinese; (2) explore the concept of awareness of disease status among cancer patients; (3) explore the impact of disease awareness on patients’ quality of life; (4) be randomized controlled studies, cohort studies, or case control studies. The following exclusion criteria were used: (1) the article was a conference abstract; (2) the full text was unavailable.

### Patient and public involvement

No patients were directly involved in this study.

### Literature retrieval and screening

We searched the following databases, PubMed, CENTRAL (Cochrane Central Register of Controlled Trials), PsycINFO, WEB OF SCIENCE, Embase, CBM (Chinese Biomedical Literature database), WANFANG database (Chinese Medicine Premier), and CNKI (China National Knowledge Infrastructure). The terms used were: neoplasm, cancer, tumor, carcinoma, disclosure, truth telling, breaking bad news, knowledge, knowing, awareness, quality of life, and QOL. Reference lists of obtained articles were hand searched and authors were contacted if articles couldn’t be easily obtained. Pairs of reviewers independently screened the literature and the third reviewer resolved any disagreements. The systematic review was registered in 2015 with PROSPERO registration number CRD42017060073. A complementary search using the above terms was performed in February 2018.

### Data extraction and management

Pairs of reviewers independently extracted the following data from included studies: first author, publication year, country, journal, the setting where the research was carried out, the time when the study began and ended, the definition of exposure in the research, study design, financial support, conflicts of interests, patients’ characteristics, and quality of life. The third reviewer resolved any disagreements.

### Primary and secondary outcome measures

The included studies used self-reported participant measures of QoL as primary or secondary end points.

#### Primary outcomes

General quality of life;

#### Secondary outcomes

1. QoL domains:
  i. physical capability (e.g. ability to perform self-care activities, mobility, and physical activities);
  ii. social capability (e.g. ability to perform work or household responsibilities and social interactions);
  iii. role function (e.g. ability to perform in daily life, amusement, and hobbies);
  iv. emotional wellbeing (e.g. levels of sadness, anxiety, depression, and/or negative affects);
  v. cognitive capacity (e.g. ability to focus attention and form/retain memories);
  vi. vitality (e.g. overall energy and fatigue);
  vii. economic ability (e.g. financial difficulty)
2. Disease-related symptoms (or both), including fatigue, pain, dyspnea, insomnia, appetite loss, and/or diarrhea.

### Assessment of risk of bias in included studies

Pairs of reviewers independently assessed risk of bias in the included studies by using the ROBINS-I assessment tool [7] for non-randomized studies, and the Cochrane risk of bias tool for randomized controlled trials. Any disagreements were resolved by discussion or consulting the third reviewer.

### Assessment of publication bias

If we included at least 10 studies in a meta-analysis, we generated funnel plots of effect estimates against their standard errors (on a reversed scale) using Review Manager software (RevMan). We assessed the potential risk of publication bias through a visual analysis of the funnel plots. Roughly symmetrical funnel plots indicated a low risk of publication bias and asymmetrical funnel plots a high risk. One should be aware that this is a rather subjective judgement and that funnel plot asymmetry might also arise from other sources and that publication bias does not always lead to asymmetry. We further attempted to avoid publication bias by searching trials registries and conference proceedings for unpublished studies. We addressed duplicate publication bias by including only one study with more than one publication. If we had doubt about whether multiple publications referred to the same data, we attempted to contact trial authors by email to resolve this issue.

### Grading of the evidence quality

Based on the results of the systematic review, the GRADE system was applied to evaluate the quality of the evidence, with results divided as follows: High quality (or A) - very confident that the real effect value is close to the estimated effect value, Moderate quality (or B) - having a moderate degree of confidence in the estimated value of the effect, and while the real value may be close to the estimated value there is still the possibility of large difference between the two groups, Low quality (or C) - limited confidence in the effect estimate and the true value may be quite different from the estimate, and Very low quality (or D) - little confidence in the effect estimate, with the true value likely to be very different from the estimate. Although evidence based on randomized controlled trails (RCT) is initially classified as high quality, confidence in such evidence may be diminished by five factors: (1) study limitations, (2) inconsistency in research results, (3) use of indirect evidence, (4) inaccurate results, and (5) publication bias. Evidence can be upgraded based on the following three factors; (1) large effect value, (2) existence of a dose-effect relationship, and (3) a possible confounding bias which may reduce efficacy.

### Data synthesis strategy

Measures of treatment effect: We analyzed continuous outcomes as standardized mean differences (SMD) between groups with 95% CIs. To assess heterogeneity, we determined statistical heterogeneity using theχ2 test. If heterogeneity was low (I2 <50%, P > 0. 05), we used the fixed effects model to calculate the combined effect. If heterogeneity was high (I2 ≥ 50%, *P* ≤ 0. 05), we used the random effects model to combine the studies. To assess reporting biases, we investigated publication and other reporting biases using funnel plots.

## Results

### Literature search

**Fig. 1:**
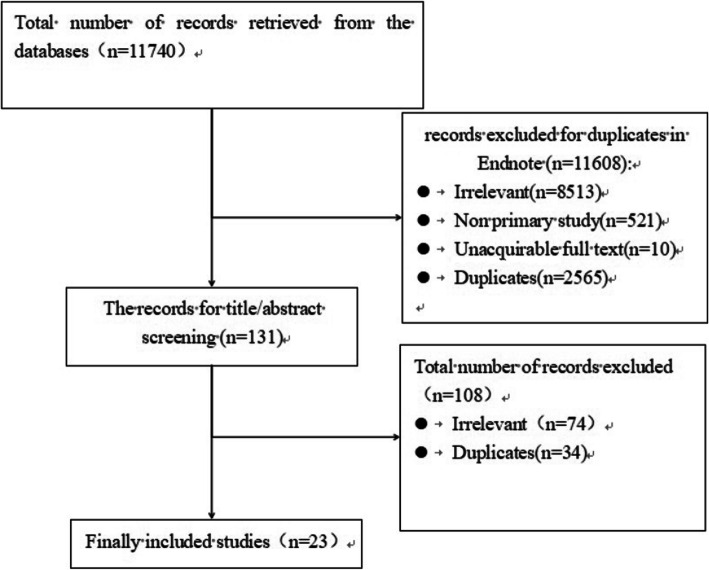
Study flow diagram

Following a comprehensive literature search, we identified and screened 11,740 references. Eleven thousand six hundred eight references were excluded based on the title and abstract. After screening the full text, a further 108 references were excluded. Following exclusions, a total of 23 references were included for further analysis. A flowchart of the search process is shown in Fig. 1.

### Overall study characteristics

The 23 included studies were all cohort studies. In all, 3322 (range 10 to 352) participants were enrolled. Detailed information on overall study characteristics are shown in Table 1.

**Table 1:** Overall study characteristics

Study origin	Journal	Country	Financial support	Length of follow-up	Sample size (exposure VS non-exposure)	Study design	Interventions (exposure VS non-exposure)	Cancer type	Quality of life assessment scale	Level of education (illiterate/primary/middle/college) (exposure VS non-exposure)	Age /years* (exposure VS non-exposure)
Noritoshi 1998 [8]	The Japan Society of Clinical Oncology	Japan	No report	1992.11 ~ 1997	23VS21	Cohort study	Truth-Disclosed VS Truth-Concealed	Gastrointestinal and Liver Cancer	Functional Living Index Cancer (FLIC)	Not report	59(54 ~ 63) VS 62(56 ~ 67)
H. Bozcuk 2001 [9]	Support Care Cancer	Turkey	Not report	Not report	56VS44	Cohort study	Aware of diagnosis VS Not aware of diagnosis	Gastrointestinal and Breast Cancer	EORTC QLQ-C30	Not report	Not report
Jianjun Zou 2006 [10]	Chinese Journal of Oncology	China	Not report	2003.1 ~ 2004.2	69VS41	Cohort study	Totally aware of the condition and partly aware of the condition VS Totally unaware of the condition	Gastrointestinal, Breast, Lung, and other Cancer	FACT-G	35/41/34/0	58 ± 12
Zhenjing Liu 2006 [11]	Journal of Psychiatry	China	No report	2005.3 ~ 2005.9	60VS64	Cohort study	Totally aware of the condition VS Totally unaware of the condition	Unknown	EORTC QLQ-C30	Not report	48 ± 12
Xiuling Wang 2006 [12]	Journal of QiLu Nursing	China	Not report	1995.1–2006.1	40VS40	Cohort study	Disclosed nursingVS Concealed nursing (disclose the truth to experiment group but conceal the truth to control group)	Liver cancer	SF-36 scale	Not report	Not report
Alexandra 2006 [13]	Progress in Palliative Care	Portugal	Not report	Not report	163VS75	Cohort study	Aware of diagnosis VS Not aware of diagnosis	Gastrointestinal, Breast, Lung, and other Cancer	EORTC QLQ-C30	Not report	59.3 ± 12.4VS 70.0 ± 9.9
Liping Zhao 2007 [14]	Journal of Nursing Science	China	Not report	2002.8 ~ 2003.1	54VS11	Cohort study	Totally aware of the condition VS Totally unaware of the condition	Liver cancer	QLS-PLC	1/10/37/17	49.3 ± 13.6
Fang Ding 2008 [15]	Chinese Nursing Research	China	Not report	2004 ~ 2006	85VS47	Cohort study	Disclosed nursing VS Concealed nursing	Unknown	GQOLI −74	Not report	18 ~ 76
Lianxue Zheng 2009 [16]	Journal of Shanxi Medical College for Continuing Education	China	Yes	2008.4 ~ 2008.7	83VS42	Cohort study	Totally aware of the condition and partly aware of the condition VS Totally unaware of the condition	Gastrointestinal cancer	EORTC QLQ-C30	0/13/103/4	57.70(28 ~ 83)
Ruihong Kong 2009 [17]	Today Nurse	China	Not report	2005.10 ~ 2007.12	115VS137	Cohort study	Totally aware of the condition VS Totally unaware of the condition	Unknown	QLQ-CCC	Not report	Not report
Zhaoxia Li 2009 [18]	Clinical Focus	China	Yes	2005 ~ 2008	87VS34	Cohort study	Totally aware of the condition VS Totally unaware of the condition	Lung cancer	EORTC QLQ-C30	39/45/37/0	51.0 ± 14.1
Ali 2009 [19]	BMC Cancer	Iran	No	2005.11 ~ 2006.4	68VS74	Cohort study	Informed of the diagnosis VS uninformed of the diagnosis	Gastrointestinal cancer	EORTC QLQ-C30	23/28/9/8 VS 55/15/3/1	50.2 ± 13.9 VS 58.2 ± 13.4
Xue Xu 2011 [20]	Master’ Thesis of Shandong University	China	Not report	2010.6 ~ 2011.4	83VS37	Cohort study	Totally aware of the condition and partly aware of the condition VS Totally unaware of the condition	Unknown	EORTC QLQ-C30	Not report	55(26 ~ 78)
Xiaoping Fan 2011 [21]	Journal of Palliative Medicine	China	Yes	2009.12 ~ 2010.07	86VS87	Cohort study	Aware of diagnosis VS Not aware of diagnosis	Gastrointestinal, Urogenital, Lung and other cancer	EORTC QLQ-C30	5/26/37/18 VS 11/38/26/12	59.35 ± 11.60 VS 62.90 ± 12.20
Yuqian Sun 2012 [22]	Chinese Journal of Behavioral Medicine and Brain Science	China	Yes	2010.12 ~ 2011.8	62VS68	Cohort study	Totally aware of the condition VS Totally unaware of the condition	Gastrointestinal cancer	EORTC QLQ-C30	Not report	54.18 ± 15.51 VS 55.73 ± 14.96
Jie Luo 2012 [23]	Cancer Research on Prevention and Treatment	China	No report	2007.6 ~ 2007.12	93VS22	Cohort study	Totally informed of the diagnosis and partly informed the diagnosis VS totally uninformed of the diagnosis	Lung cancer	EORTC QLQ-C30	0/34/63/18	#
Lina Wang 2013 [24]	Journal of Nurses Training	China	Not report	2012.1 ~ 2012.12	89VS98	Cohort study	Totally aware of the condition VS Totally unaware of the condition	Gastrointestinal cancer	EORTC QLQ-C30	Not report	30.9 ± 11.3 VS 31.1 ± 11.0
Liping Fu 2013 [25]	Chinese Journal of Gerontology	China	Not report	2007 ~ 2012	100VS100	Cohort study	Totally aware of the condition VS Totally unaware of the condition	Lung cancer	EORTC QLQ-C30	Not report	73.5 ± 15.8
Zaili Feng 2014 [26]	Anti-Tumor Pharmacy	China	Not report	Not report	352VS68	Cohort study	Informed of the diagnosis VS uninformed of the diagnosis	Gastrointestinal, Breast, Lung, and other Cancer	Jiacheng Li Foundation for Hospice Plan Quality Life Scale)	Not report	48.0 ± 19.1 VS 49.7 ± 18.2
Yuanling Li 2014 [27]	International Journal of Nursing	China	Not report	2011.12 ~ 2013.12	30VS30	Cohort study	Disclosed nursing VS Concealed nursing	Liver cancer	SF-36 scale	Not report	54.3 ± 19.4 VS 51.4 ± 17.9
Nobuhisa 2015 [28]	American Journal of Hospice & Palliative Medicine	Japan	Not report	2004.4 ~ 2008.3	15VS10	Cohort study	Informed VS uninformed	Gastrointestinal, Liver and Breast Cancer	STAS-J scale	Not report	72.8 + 11.8
Bo Yang 2015 [29]	Hainan Medical Journal	China	Not report	2012.9 ~ 2013.9	30VS63	Cohort study	Totally aware of the condition VS Totally unaware of the condition	Gastrointestinal, Breast, Lung, and other Cancer	EORTC QLQ-C30	9/21/0/0	69.80 ± 5.11 VS 71.95 ± 5.45
Ruifen Zhang 2016 [30]	Journal of Clinical Medical Literature	China	Not report	2005.2–2005.10	36VS36	Cohort study	Disclosed nursing VS Concealed nursing	Liver cancer	SF-36 scale	Not report	49.5 ± 0.8 VS 48.1 ± 1.9

### Risk of bias in included studies

Included studies were assessed for risk of bias using the ROBINS-I assessment tool. For each trial the risk of bias is detailed in Table 2.

**Table 2:** Risk of bias summary: review authors’ judgements about each risk of bias item for each included study

Study ID	1.Bias due to confounding	2.Bias in selection of participants into the study	3.Bias in classification of interventions	4.Bias due to deviations from intended interventions	5.Bias due to missing data	6.Bias in measurement of outcomes	7.Bias in selection of the reported result	overall risk of bias
Ali 2009 [19]	***	****	****	****	****	****	^a^	***
Xiaoping Fan 2011	***	****	****	**	***	****	^a^	**
Yuanling Li 2014 [27]	***	****	****	****	****	****	^a^	***
Jianjun Zou 2006 [10]	**	****	****	****	****	****	^a^	***
Jie Luo 2012 [23]	**	****	****	****	****	****	^a^	**
Zhenjing Liu 2006 [11]	**	****	*	****	****	****	^a^	*
Noritoshi 1998 [8]	**	****	****	****	***	****	****	**
Nobuhisa 2015 [28]	**	****	****	****	*	****	^a^	*
Liping Zhao 2007 [14]	**	****	****	****	****	****	^a^	**
Lianxue Zheng 2009 [16]	*	****	****	****	****	****	^a^	*
Ruihong Kong 2009 [17]	*	****	****	****	*	****	^a^	*
Zaili Feng 2014 [26]	**	****	****	****	****	****	^a^	**
Xue Xu 2011 [20]	***	****	****	****	****	****	^a^	****
Lina Wang 2013 [24]	****	****	****	****	***	****	^a^	***
Fang Ding 2008 [15]	**	****	****	****	****	****	^a^	**
Zhaoxia Li 2009 [18]	**	****	***	****	****	****	^a^	**
Bo Yang 2015 [29]	****	****	***	****	****	****	^a^	***
Yuqian Sun 2012 [22]	**	****	***	****	****	****	^a^	**
Alexandra 2006 [13]	***	****	****	****	****	****	^a^	***
H. Bozcuk 2001 [9]	***	****	****	****	****	****	^a^	***
Liping Fu 2013 [25]	**	****	***	****	****	****	^a^	**
Xiuling Wang 2006 [12]	**	****	****	**	****	****	^a^	**
Ruifen Zhang 2016 [30]	**	****	****	**	****	****	^a^	**

**** Low

*** Moderate

** Critical

^a^No information

### Meta-analysis results

#### Overall quality of life

**Fig. 2:**
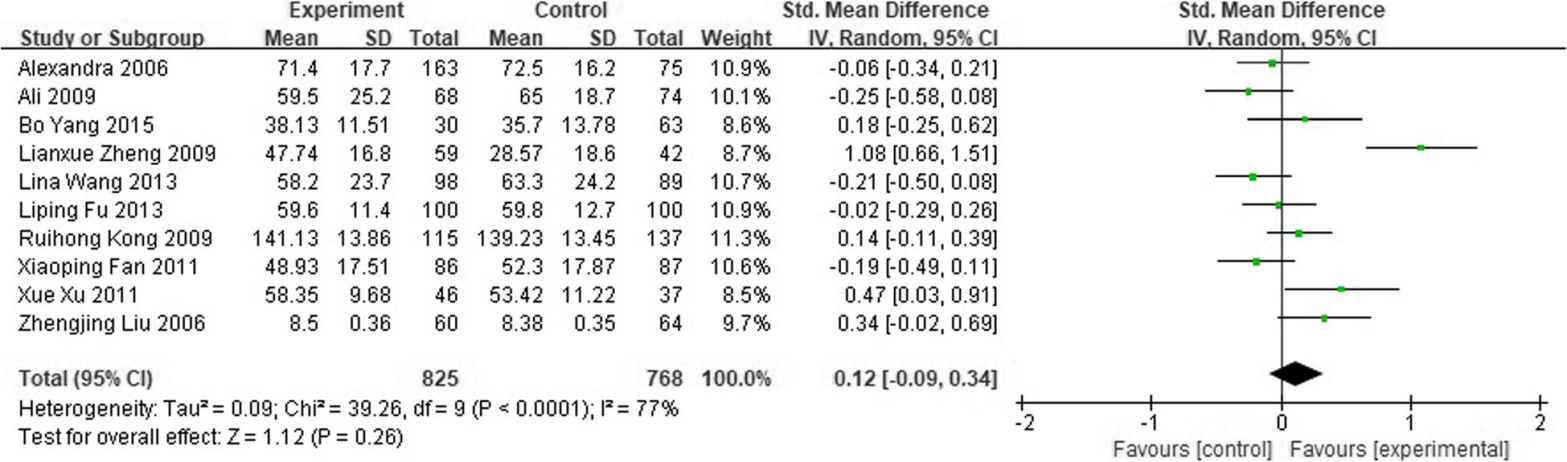
Forest plot of overall quality of life between totally informed of diagnosis and totally uninformed of diagnosis in cancer patients

**Fig. 3:**
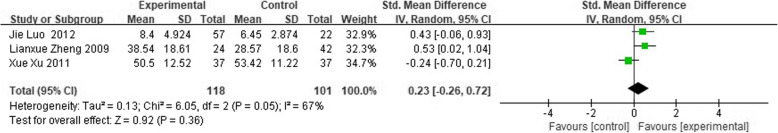
Forest plot of overall quality of life between partly informed of diagnosis and totally uninformed of diagnosis in cancer patients

There was no difference in the change in QoL from baseline between totally informed and uninformed of diagnosis in 1593 study patients (SMD 0.12; 95% CI-0.09 to 0.34), and no difference between partly informed and uninformed of diagnosis in 219 participants (SMD 0.23; 95% CI-0.26 to 0.72). Details shown in Figs. 2 and 3.

#### Role function

Meta-analyses comparing totally informed with control intervention showed no differences in role function among 1250 patients. The same result was seen with patients partly informed of diagnosis. See Table 3 for detailed information.

**Table 3:** Overall Meta-analysis summary between Totally informed of diagnosis and Uninformed of diagnosis in cancer patients

Outcome or subgroup	Participants	Std. Mean Difference (IV, Random, 95% CI)	*P* value
General Quality of Life	1593	0.12 [− 0.09, 0.34]	0.26
Function domains			
Role Function	1250	0.17 [−0.05, 0.39]	0.13
Cognitive Activity	1150	0.61 [− 0.06, 1.28]	0.08
Vitality	212	2.22 [0.11, 4.33]	0.04
Emotional Function	1793	0.13 [−0.20, 0.47]	0.43
Social Function	2045	0.58 [0.11, 1.05]	0.02
Physical Function	1733	0.03 [−0.26, 0.32	0.83
Disease-related symptoms			
Nausea and Vomiting	1250	−0.13[− 0.46, 0.20]	0.45
Pain	1541	−0.24[− 0.61, 0.14]	0.22
Dyspnea	1250	−0.01[− 0.12, 0.10]	0.88
Fatigue	1250	0.07 [−0.23, 0.38]	0.63
Diarrhea	1250	−0.03[− 0.21, 0.15]	0.77
Constipation	1250	0.04 [−0.12, 0.20]	0.62
Appetite Loss	1250	0.06 [−0.05, 0.17]	0.30
Insomnia	1250	0.08 [−0.05, 0.21]	0.21

#### Cognitive activity

We found no significant effect on cognitive activity from totally informing cancer patients of diagnosis. See Table 3 for detailed information.

#### Physical function

No difference in scores was observed between totally informed and uninformed of diagnosis groups in 1150 cancer patients. See Table 3 for detailed information.

#### Social function

**Fig. 4:**
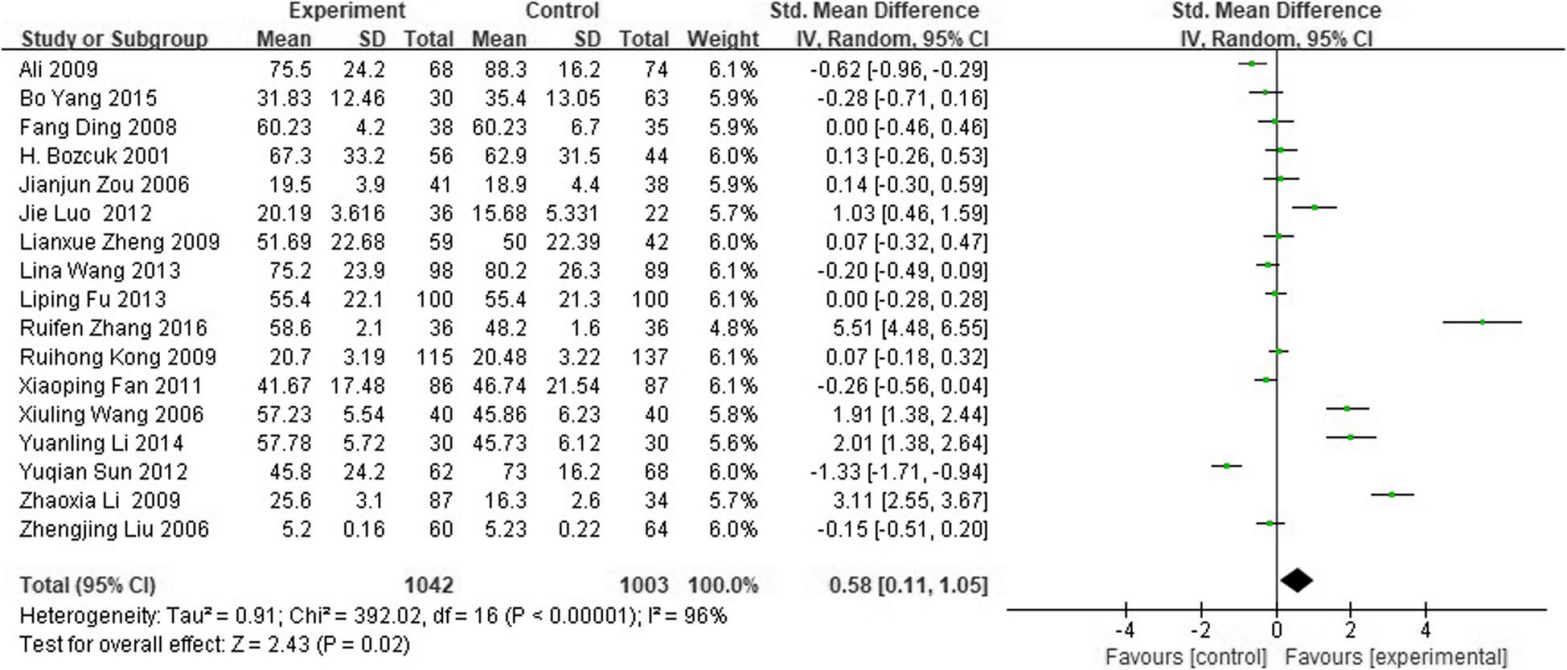
Forest plot of social function between totally informed of diagnosis and totally uninformed of diagnosis in cancer patients

**Fig. 5:**
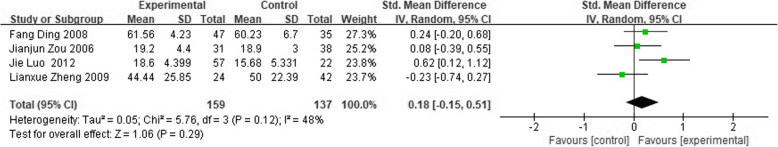
Forest plot of social function between partly informed of diagnosis and totally uninformed of diagnosis in cancer patients

**Fig. 6:**
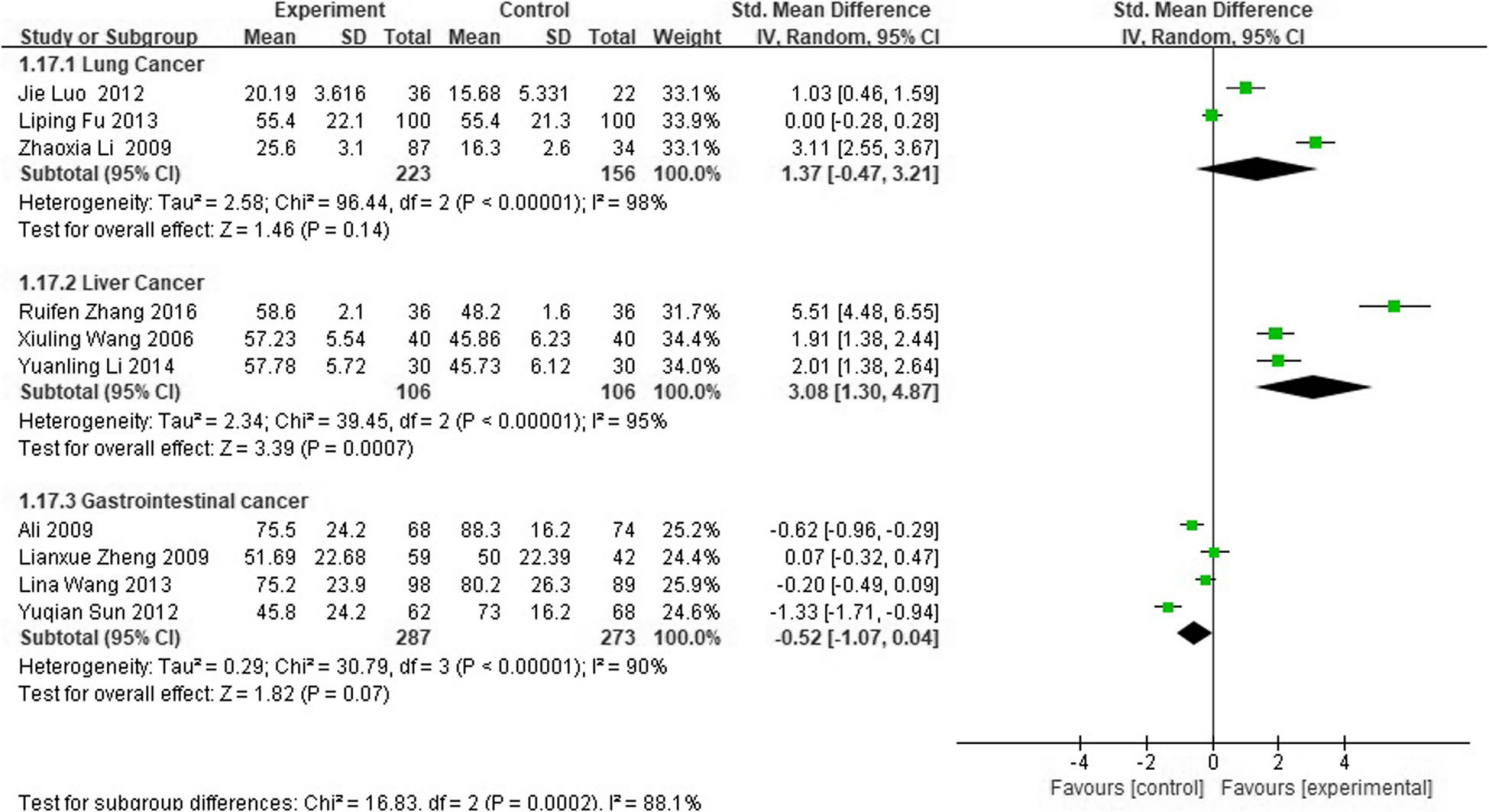
Subgroup analysis based on cancer types in social function between partly informed of diagnosis and totally uninformed of diagnosis in cancer patients

Compared to patients uninformed of diagnosis, totally informed patients did better, and their social function was significantly affected among 2130 cancer patients (SMD 0.63; 95% CI 0.18 to 1.09). Subgroup analysis based on cancer types showed that there was no difference in lung and gastrointestinal cancer patients (*P* > 0.05), while in liver cancer, patients totally informed of diagnosis did better than uninformed patients (SMD 3.08; 95%CI 1.30 to 4.87). No difference was seen between the partly and totally uninformed of diagnosis groups (SMD 0.18; 95% CI − 0.15 to 0.51) in 296 patients. See Figs. 4, 5 and 6 for forest picture.

#### Vitality

**Fig. 7:**
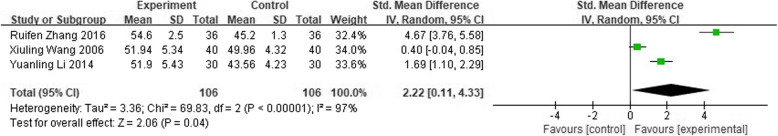
Forest plot of vitality between totally informed of diagnosis and totally uninformed of diagnosis in cancer patients

Totally informed were significantly better than uninformed of diagnosis in role function among 212 cancer patients (SMD 2.22; 95%CI 0.11 to 4.33). No information on partly informed versus totally uninformed patients was found for use in this study. More information is shown in Fig. 7.

#### Emotional function

No difference was seen between the totally and partly informed diagnosis groups compared to totally uninformed groups. See Table 3 for detailed information.

#### Economic difficulty

**Fig. 8:**
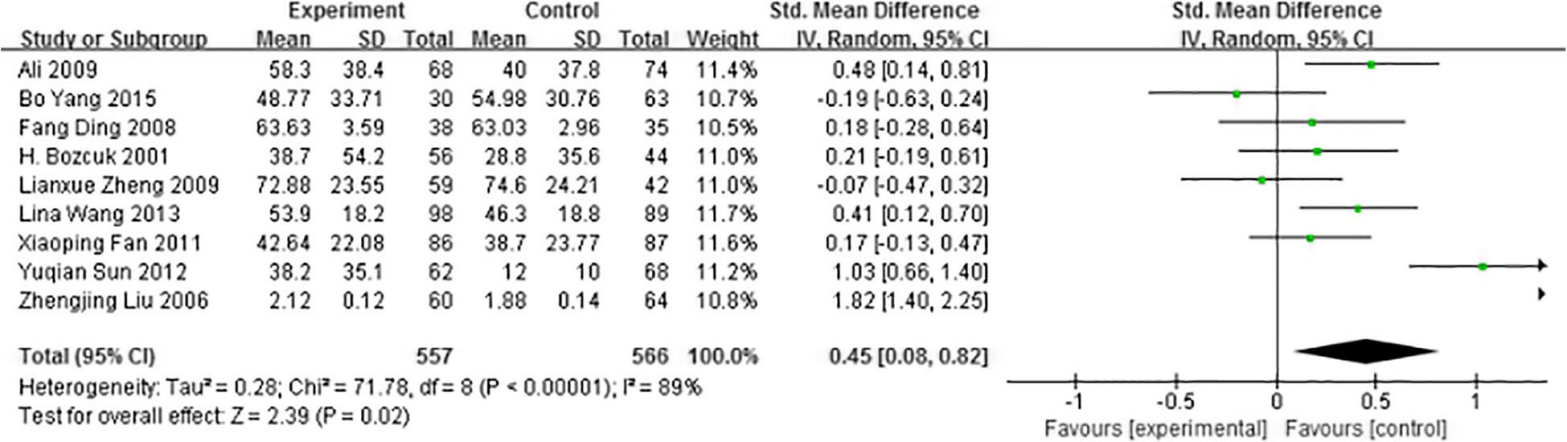
Forest plot of Economic difficulty between totally informed of diagnosis and totally uninformed of diagnosis in cancer patients

We observed that in terms of economic function, totally informed performed significantly worse than uninformed of diagnosis groups in 1123 participants when looking at the change in scores across instruments from baseline to follow-up (SMD 0.45; 95%CI 0.08 to 0.82). Totally informed of diagnosis patients more often felt economic difficulty than those uninformed of diagnosis. See Fig. 8 for detailed information.

#### Disease-related symptoms

We observed no significant effect between totally informed and uninformed of diagnosis groups in assessments of fatigue, pain, dyspnea, diarrhea, constipation, appetite loss, insomnia, nausea, and vomiting. Details shown in Tables 3 and 4.

**Table 4:** Overall Meta-analysis summary between partly informed of diagnosis and totally uninformed of diagnosis in cancer patients

General Quality of Life	219	0.23 [− 0.26, 0.72]	0.36
Function domains			
Physical Function	286	0.01 [−0.22, 0.25]	0.93
Social Function	296	0.18 [−0.15, 0.51]	0.29
Emotional Function	296	−1.24[−2.75, 0.26]	0.11
Disease-related symptoms			
Pain	217	−0.15[−0.42, 0.13]	0.30

### Grading of evidence quality

Results based on systematic reviews were graded low and very low. Details in Table 5.

**Table 5:** Summary of findings for the main comparison

Totally informed of diagnosis versus uninformed of diagnosis				
Patient: cancer patients				
Intervention: totally informed of diagnosis				
Comparison: uninformed of diagnosis				
Outcomes	Sample Size (Number + Study Design)	Evidence Grade	Relative Effect (95% CI)	Prospective Absolute Effect (95%CI)
General Quality of Life	1593 (10 cohort studies)	Very Low^1^ ⊕ ○○○	SMD 0.12 [− 0.09, 0.34]	SMD 0.12 SD higher (− 0.09 lower to 0.34 higher)
Role Functioning	1250 (9 cohort studies)	Low ⊕ ⊕ ○○	MD 0.17 [−0.05, 0.39]	MD 0.17 higher (− 0.05 lower to 0.39 higher)
Cognitive Activity	1150 (8 cohort studies)	Very Low^2^ ⊕ ○○○	SMD 0.61 [− 0.06, 1.28]	SMD 0.61 higher (− 0.06 lower to 1.28 higher)
Vitality	212 (3 cohort studies)	Very Low^2 3 4^ ⊕ ○○○	SMD 2.22 [0.11, 4.33]	SMD 2.22 higher (0.11 lower to 4.33 higher)
Emotional Function	1793 (14 cohort studies)	Very Low ^5^ ⊕ ○○○	SMD 0.13 [−0.20, 0.47]	SMD 0.13 higher (−0.20 lower to 0.47 higher)
Social Function	2045 (17 cohort studies)	Very Low ^6^ ⊕ ○○○	SMD 0.58 [0.11, 1.05]	SMD 0.58 higher (0.11 lower to 1.05 higher)
Physical Function	1733 (13 cohort studies)	Low ^7^ ⊕ ⊕○○	SMD 0.03 [−0.26, 0.32]	SMD 0.03 higher (− 0.26 lower to 0.32 higher)
Nausea and Vomiting	1250 (9 cohort studies)	Very Low ^8^ ⊕ ○○○	SMD − 0.13 [− 0.46, 0.20]	SMD − 0.13 higher (− 0.46 lower to 0.20 higher)
Pain	1541 (13 cohort studies)	Very Low^9^ ⊕ ○○○	SMD − 0.24 [− 0.61, 0.14]	SMD − 0.24 higher (− 0.61 lower to 0.14 higher)
Dyspnea	1250 (9 cohort studies)	Low ⊕ ⊕ ○○	SMD − 0.01 [− 0.12, 0.10]	SMD − 0.01 higher (− 0.12 lower to 0.10 higher)
Fatigue	1250 (9 cohort studies)	Very Low^10^ ⊕ ○○○	SMD 0.07 [− 0.23, 0.38]	SMD 0.07 higher (− 0.23 lower to 0.38 higher)
Financial Difficulty	1123 (9 cohort studies)	Very Low^8^ ⊕ ○○○	SMD 0.14 (0.01 ~ 1.47)	SMD 0.14 higher (0.01 lower to 1.47 higher)
Diarrhea	1250 (9 cohort studies)	Very Low^11^ ⊕ ○○○	SMD − 0.03 [− 0.21, 0.15]	SMD − 0.03 higher (− 0.21 lower to 0.15 higher)
Constipation	1250 (9 cohort studies)	Low ⊕ ⊕ ○○	SMD 0.04 [− 0.12, 0.20]	SMD 0.04 higher (− 0.12 lower to 0.20 higher)
Appetite Loss	1250 (9 cohort studies)	Low ⊕ ⊕ ○○	SMD 0.06 [− 0.05, 0.17]	SMD 0.06 higher (− 0.05 lower to 0.17 higher)
Insomnia	1250 (9 cohort studies)	Low ⊕ ⊕ ○○	SMD 0.08 [− 0.05, 0.21]	SMD 0.06 higher (− 0.05 lower to 0.17 higher)
Partly informed of diagnosis versus uninformed of diagnosis				
Patient: cancer patients				
Intervention: partly informed of diagnosis				
Comparison: uninformed of diagnosis				
General Quality of Life	219 (3 cohort studies)	Very Low^12^ ⊕ ○○○	SMD 0.23 [− 0.26, 0.72]	SMD 0.23 higher (− 0.26 lower to 0.72 higher)
Pain	217 (3 cohort studies)	Very Low^3 4^ ⊕ ○○○	SMD − 0.15 [− 0.42, 0.13]	MD − 0.15 higher (− 0.42 lower to 0.13 higher)
Physical Function	286 (4 cohort studies)	Very Low^3 4^ ⊕ ○○○	SMD 0.01 [− 0.22, 0.25]	SMD 0.01 higher (− 0.22 lower to 0.25 higher)
Social Function	296 (4 cohort studies)	Very Low^3 4^ ⊕ ○○○	SMD 0.18 [− 0.15, 0.51]	SMD 0.18 higher (− 0.15 lower to 0.51 higher)
Emotional Function	296 (4 cohort studies)	Very Low^3 4^ ⊕ ○○○	SMD − 1.24 [− 2.75, 0.26]	SMD − 1.24 higher (− 2.75 lower to 0.26 higher)

*CI* Confidence interval, *SMD* Standardized mean difference

GRADE Working Group grades of evidence

High quality: Further research is very unlikely to change our confidence in the estimate of effect

Moderate quality: Further research is likely to have an important impact on our confidence in the estimate of effect and may change the estimate

Low quality: Further research is very likely to have an important impact on our confidence in the estimate of effect and is likely to change the estimate

Very low quality: We are very uncertain about the estimate

Reasons for downgraded:

1. The confidence interval’ overlaps were low and I^2^ was 70%

2. The confidence interval’ overlaps were low and I^2^ was 97%

3. The sample sizes were fewer than 300 participants included in the total

4. The 95% confidence interval was too wide

5. The confidence interval’ overlaps were low and I^2^ was 91%

6. The confidence interval’ overlaps were low and I^2^ was 96%

7. The confidence interval’ overlaps were low and I^2^ was 88%

8. The confidence interval’ overlaps were low and I^2^ was 89%

9. The confidence interval’ overlaps were low and I^2^ was 92%

10. The confidence interval’ overlaps were low and I^2^ was 86%

11. The confidence interval’ overlaps were low and I^2^ was 60%

12. The confidence interval’ overlaps were low and I^2^ was 67%

### Publication bias

**Fig. 9:**
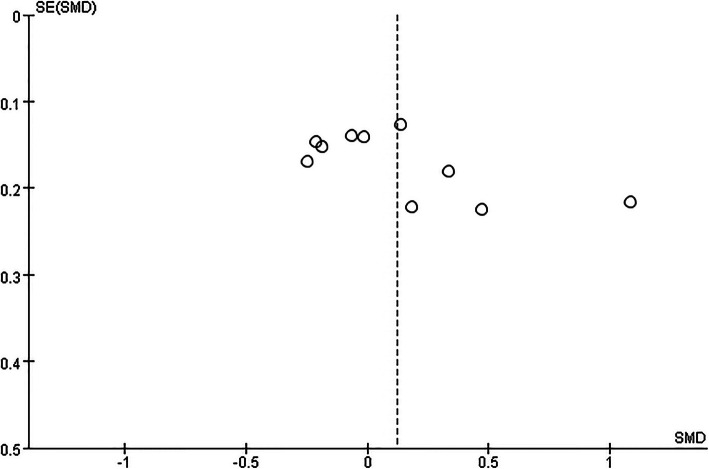
Funnel plot in the meta-analysis of overall quality of life between totally informed of diagnosis and totally uninformed of diagnosis in cancer patients

Because we included 10 studies in the meta-analysis of overall quality of life between totally informed and totally uninformed of diagnosis cancer patients, we generated a funnel plot of effect estimates against their standard errors (on a reversed scale) using Review Manager software (RevMan). The funnel plot was nearly symmetrical and every meta-analysis exited negative and positive results, which meant that there is little possibility of publication bias in this study. See Fig. 9 for detailed information.

## Discussion

### Summary of main results

We included 23 trials with 3322 participants distributed over totally informed, partly informed, and uninformed of diagnosis groups. Conference abstracts and studies whose full text was unavailable were excluded. Almost all the included studies were of low quality, among which 20 studies had an existing bias due to various confounding factors such as age and degree of education, and only 5 had an adjusting analysis. The 3 other studies were bias-free due to the consistency of their confoundings and baselines. Results based on systematic reviews were graded low and very low. The main reasons for their downgrading were that the confidence interval overlaps were low and I^2^ was larger than 50%, sample sizes had fewer than 300 participants included in the total, and the 95% confidence interval was too wide.

Through meta-analysis, cancer patients who were totally informed or uninformed of the diagnosis had no differences in either their general quality of life and symptoms of fatigue, pain, dyspnea, insomnia, appetite loss, and diarrhea (*P* > 0.05). There was also no difference in the physical function, role function, cognitive activity, and emotional function, of the groups (*P* > 0.05). However, in terms of vitality and social function, totally informed patients did better than uninformed patients. Subgroup analysis based on cancer types showed that liver cancer patients who were totally informed of their diagnosis did better than those uninformed in social function, but informed patients seemed to get higher scores in financial difficulty. Between the partly informed and uninformed groups, no differences were found in general quality of life, function domains, and disease-related symptoms (P > 0.05).

### Implications for practice

Cancer is a special concern around the world and a patients’ quality of life is an important aspect in their therapeutic journey [31–34]. The issue of whether cancer patients should be informed of their diagnosis has long been debated [35]. Some people contend that telling the truth to them and their relatives upholds their right to know, while others would say that white lies can ease worries and help patients’ psychological defense [9, 19, 22, 25, 35]. Our results showed that there is no significant impact on health-related quality of life in cancer patients between the patient being fully informed, partially informed, or completely uninformed of their cancer diagnosis. This indicates that physicians could inform patients and educate them, which would help them understand their cancer and get the families, patients, and doctors in charge together to make personalized and systematic therapy plans and accurately evaluate prognosis [8]. Concealing the truth might render patients’ suspicious and gloomy, potentially leading to depression that could promote tumor progression. When exposing patients to the truth, it would be better for the clinicians to educate patients and their families separately. This is because patients need more knowledge about the cancer to fight against it bravely and optimistically, while their families need more patience and confidence to help support the patients [8, 21, 28, 36]. This may be a future research direction in clinical practice to help improve cancer patients’ education.

### Implications for research

This systematic review and meta-analysis of 23 trials examined whether a cancer patients level of information of their diagnosis affected their health-related quality of life. It provides evidence that a patients’ knowledge of their diagnosis may have no effect on the general quality of life or on their symptoms of fatigue, pain, dyspnea, insomnia, appetite loss, physical function, role function, cognitive activity, and emotional function, and may in fact have beneficial effects in terms of vitality and social function.

Further research is required to evaluate the best way to tell patients the truth. Following on from the work of Ruifen Zhang 2016 [30], Fang Ding 2008 [15], and Xiuling Wang 2006 [12], we can suppose that delivering the truth to cancer patients combined with comprehensive nursing, especially mental health nursing, could be beneficial to their quality of life, however, whether it actually makes difference is still unknown. It would be helpful if there were more research on specific cancer types, such as lung, stomach, liver, colon, and breast, to determine if different outcomes on QoL are seen with different cancer types.

Quality of life is an important measure of cancer survival, but because of the quantities of scales, heterogeneity is large, which makes comparing findings between trials extremely difficult. To overcome this problem, health-related quality of life scales should be standardized in the future. Our results were consistent with the findings of Aggarwal A [7].

### Strengths and limitations of this study

The results of this study will give clinicians and patients’ family some enlightenment on communication with cancer patients. Our conclusion relies on both the quality and quantity of the original studies available for review, and the low-quality evidence in our studies may affect any extrapolation of our conclusion. Because our research went on for a long period of time, we conducted a complementary search to avoid missing the latest original studies. The biggest limitation in our study was the different health-related quality of life scales which increased heterogeneity and made comparing findings between trials extremely difficult. However, we were still able to analyze these continuous outcomes as standardized mean differences (SMD) between groups with 95% CIs. To assess heterogeneity, we determined statistical heterogeneity using the χ2 test. If heterogeneity was low (I2 <50%, P > 0. 05), we used the fixed effects model to calculate the combined effect and if heterogeneity was high (I2 ≥ 50%, *P* ≤ 0. 05), we used the random effects model to combine the studies. The sub-subgroups were then divided into lung, liver, and gastrointestinal cancer to decrease heterogeneity.

## Conclusion

Informing cancer patients about their diagnosis may not have a detrimental effect on their quality of life, but more studies based on high quality evidence are still required.

## Data Availability

No additional data is available.

## Abbreviations

EORTC: European Organization for Research and Treatment of Cancer
GRADE: Grading of Recommendation, Assessment, Development and Evaluation
NOS: Newcastle-Ottawa Scale
SMD: Standardized mean difference
